# Technology-Supported Behavior Change—Applying Design Thinking to mHealth Application Development

**DOI:** 10.3390/ejihpe14030039

**Published:** 2024-03-07

**Authors:** Ramona Schweitzer, Stephan Schlögl, Marco Schweitzer

**Affiliations:** 1 Department Digital Business & Software Engineering, MCI|The Entrepreneurial School, Universitätsstrasse 15, 6020 Innsbruck, Austria; 2 Department Management, Communication & IT, MCI|The Entrepreneurial School, Universitätsstrasse 15, 6020 Innsbruck, Austria; 3 Division for Digital Health and Telemedicine, UMIT TIROL—Private University for Health Sciences and Health Technology, Eduard-Wallnöfer-Zentrum 1, 6060 Hall in Tirol, Austria

**Keywords:** mHealth, behavioral change, design thinking, user-centered design

## Abstract

Non-communicable diseases are the leading cause of global deaths. The risk of their development and progression is increased by modifiable behavioral risk factors. Yet, despite the known benefits of primary and secondary prevention, people often do not follow recommendations for a healthier lifestyle. To this end, mobile health (mHealth) applications offer features for behavioral interventions. Yet, reported user engagement is often low. The objective of the work presented in this article is thus to evaluate the suitability of Design Thinking (DT) as a means to inform the development of an mHealth application that helps increase long-term engagement, and consequently supports individuals in sustainably changing their lifestyle. Applying the DT approach, key user needs and challenges were investigated and used to design a first low-fidelity mHealth application prototype. Think-Aloud analysis, task completion, and post-test interviews were then used to evaluate the prototype and generate early-stage insights. Subsequently, a structured, retrospective analysis of this process, evaluating the insight-generation potential of each step in the DT process cycle, was used to reflect on its suitability to inform mHealth application development. The respective results highlight (1) the distinct value of the DT method, particularly in the early stages of a development project; (2) the strong need for interdisciplinary collaboration in such projects, so as to capture realistic end-user requirements and improve the overall effectiveness of the application design; and (3) the significance of integrating behavioral change theories into the design of mHealth applications, in order to promote long-term engagement.

## 1. Introduction

Non-communicable diseases (NCDs) (i.e., chronic diseases like cardiovascular diseases, different types of cancer, chronic respiratory diseases, or diabetes) count as the leading cause of death today. In 2016, 71% of all deaths on the globe were already attributed to an NCD [1], and according to the World Health Organization (WHO), this figure has risen to 74% in 2022 (online: https://www.who.int/news-room/fact-sheets/detail/noncommunicable-diseases [accessed: 24 July 2023]).

The risk of the development and progression of an NCD is increased by modifiable behavioral risk factors, such as physical inactivity, unhealthy diet, bad stress management, and tobacco use [2,3,4]. A healthy lifestyle, on the other hand, can help reduce the respective risk factors. Yet, despite the knowledge about the benefits of primary and secondary prevention mechanisms, there is strong evidence suggesting that the world’s population does not meet the recommended behavior [1]. For example, almost one-third of adults worldwide are insufficiently active [5]. This is not only crucial with respect to an individual’s quality of life and a population’s overall survival rate, but it also leads to a substantial economic burden. Physical inactivity alone is responsible for worldwide costs of at least USD 67.5 billion per year, caused by healthcare expenditure and productivity losses [6]. Thus, there is an urgent need for effective strategies to enhance a healthy lifestyle for people. To this end, a promising way to prevent and control non-communicable diseases is seen in technology-supported health-related interventions that aim to positively affect lifestyle behavior.

### 1.1. Technology-Supported Health-Related Interventions

New technologies, in particular mobile health applications [7,8,9,10,11], offer great potential in increasing the effectiveness of and adherence to health-related interventions. The WHO defines mobile health (mHealth) as *“medical and public health practice supported by mobile devices, such as mobile phones, patient monitoring devices, personal digital assistants (PDAs), and other wireless devices”* [12]. mHealth applications (i.e., apps) usually include and combine various digital instruments and methods, aiming to improve and maintain a user’s health [13]. For this, mobile devices offer various features for behavioral interventions. They are also highly valued by their owners, which fosters technology engagement, and they remain with their users nearly throughout the whole day [14], which helps with the implementation of interventions, bringing them to real-life contexts where decisions about health behavior are usually made and where diverse barriers to behavioral change may thus be encountered [9]. Also, mobile devices facilitate the collection and potential sharing of behavioral and health-related data with healthcare professionals. For example, a smartphone can automatically collect data on a user’s movement, emotion, and engagement, which consequently allows for the automated tracking of health-related behaviors over time [15].

Another advantage may be seen in the fact that, for users, mHealth applications usually provide a low-threshold, cost-effective, feasible and convenient form of interaction [11,16], which increases user engagement and, consequently, the effectiveness of interventions. For this, cost-effectiveness not only relates to the financial aspect, which certainly helps with the penetration across different socioeconomic groups [17], but also considers so-called opportunity costs, which may keep people from engaging in other valued activities [18,19]. In general, it has been found that the attitude towards mobile applications helping people in adopting a healthy lifestyle is positive [9]. Therefore, mHealth solutions have increasingly been used by health systems and health workers to provide services and communicate with patients and clients [20]. The health conditions and clinical areas being addressed by apps have also strongly expanded over time, from 13 distinct clinical areas reported in 2014 to 48 in 2019 [21]. The number of mHealth apps, including health and fitness, as well as medical apps, has also increased in recent years, so that in 2017, more than 325,000 mHealth apps were available on all major app stores, 25% of which had been added after 2016 [22].

### 1.2. The Challenge of Dropouts

Based on the above discussion, one may certainly argue that mobile apps are effective in supporting healthy behavior, such as increasing physical activity and fitness, decreasing sedentary behavior, or promoting a healthy diet [16,18,23]. However, most of these positive effects have only been observed in the short-term, after which, high dropout rates are reported [18,23,24]. Especially after the first few weeks of an intervention, participant engagement declines [9,11], which is an important factor, since an app’s efficacy is significantly correlated with its usage [8]. As dropout rates with mHealth applications are comparable to those seen with conventional interventions (i.e., those without the use of mobile devices), it is suggested to use behavior maintenance strategies [25] to foster intervention effectiveness. That is, interventions to improve health behavior need to be designed based on an explicit theoretical foundation, which implements relevant theories of behavioral change [26]. On the other hand, to promote long-term engagement and intervention efficacy, the development of the proposed evidence-based treatments (EBTs) should be based on strategies that ensure a high usability of the employed technology [21,26,27]. Here a user-centered [28,29] or human-centered [30] design approach is often recommended. Said approach is grounded in continuous user engagement, where, from the early stages of development on, users have to be placed at the center of the technology/software design process. This fosters the overall quality of a solution by improving user experience and productivity, and reducing the risk of harm, discomfort, and stress. Furthermore, it considers variable user needs, characteristics, and capabilities and, therefore, increases the technology’s accessibility [30]. Relevant steps in such a design process include activities (1) to understand and specify the context of use, (2) to specify the user requirements, (3) to then produce design solutions that meet these specified user requirements, and finally, (4) to evaluate respective solutions with users [30].

### 1.3. Building User-Centered mHealth Applications

Various stakeholders have to be considered when designing, developing, and deploying healthcare applications [31], since it is particularly the continuous use of these apps that significantly impacts the effectiveness of the therein-transferred medical intervention. Thus, also the health technology sector, similar to other domains, calls for more user-centered, participatory design and development approaches so as to increase the success of interventions. A key goal of these user-centered approaches is the generation of a better, more in-depth understanding of the different perspectives, goals, and concerns that are inherent to users’ distinct needs, be it patients or clinicians [32]. To this end, the method of Design Thinking (DT) holds a strong potential to close this gap between intervention design and tool implementation, as it incorporates user needs throughout the entire design process [33]. It has been shown that, compared to traditional development approaches, the integration of DT can lead to higher usability, especially user satisfaction, and acceptability [33].

**Figure 1:**
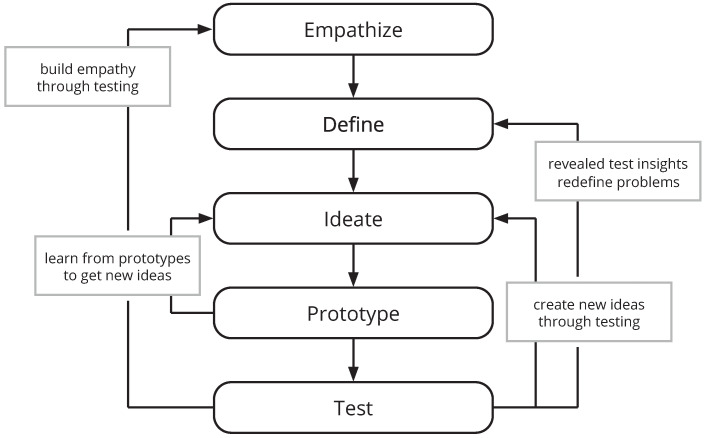
The core activities of Design Thinking and their output.

The core activities in DT are emphasized by the five DT modes [34] (cf. Figure 1). The first mode, referred to as *Empathize*, promotes the continuous engagement with people who are affected by the interaction with a product or service. Their behavior is observed, which helps in learning their values and uncovering their needs. For this, the first DT mode represents the initial understanding of the user and thus provides directions for the subsequent phases of the process. Next, the second mode, referred to as *Define*, addresses the actionable problem statements produced in the preceding mode by producing unique design visions. Following this, the *Ideate* mode generates radical design alternatives. Here, the purpose is to explore a wide solution space, so as not to end up caught in just one concept or outcome. Actual trial versions of a system or its components are then developed during the *Prototype* mode, which often also triggers new ideas. Finally, the *Test* mode evaluates the produced prototypes with actual users and, through this, generates valuable feedback and further insights.

The DT components most applicable to healthcare are the creation of empathy, collaboration, and rapid prototyping [35]. Empathy is created through a detailed understanding of users’ health-related needs, values, and desires. It usually requires an initial analytical phase aimed at reviewing existing literature and contextual data so as to identify particular user groups [35]. The subsequent creation of concrete personas helps circumvent a certain developer bias, which often causes engineers to build solutions for users who are similar to themselves. To this end, collaboration among different disciplines is important to address complex, healthcare-related domain issues [35]. In other words, a clear conceptualization including an explicit understanding of user types and perspectives across different intervention phases is required. This can be achieved by continuously involving clinicians with expertise in the application of evidence-based treatment in community settings [26]. Furthermore, it has been shown that the cooperation with credentialed experts during the whole development process can lead to higher engagement in the use of the resulting health behavior change applications [8,9].

Finally, DT’s proposed rapid prototyping stage allows for the testing of multiple alternative hypotheses and strategies. It also helps to discover unintended consequences and potential challenges of the envisioned health-related interventions [35]. Also, when carefully planned and managed, rapid prototyping can be rather efficient [36], which is especially important when working with physicians since their time capacities are often very limited [18,37]. Paper prototyping is considered particularly effective here, for it prevents the exclusion of stakeholders with insufficient prototyping skills [38,39], yet still allows for visually communicating design ideas so as to gain a shared understanding of a problem space [40]. Yet, despite these advantages, and although a review of 24 interventions across different health conditions has demonstrated that DT is feasible and applicable to building applications for multiple healthcare domains [33], the respective methods were only considered in approximately one-third of the mHealth studies reviewed by Maramba et al. [21].

One reason for this lack of use may be found in some of DT’s limitations and challenges. The thorough user research, iterative ideation, prototyping, and testing phases, for example, make for a lengthy endeavor (online: https://www.interaction-design.org/literature/article/5-stages-in-the-design-thinking-process [accessed: 23 February 2024]). Smaller organizations or those with limited resources might thus struggle to support the full DT cycle. Additionally, like any user-centered approach, DT is vulnerable to biases. These biases can manifest during the empathy phase where designers might unconsciously project their own experiences or worldviews onto the user [41]. To counteract this, it is critical to actively cultivate diverse perspectives within the team. To this end, another potential pitfall of DT concerns the risk of neglecting relevant stakeholders so that the resulting design solutions may inadvertently have negative environmental consequences or fail to address social justice concerns [42]. Hence, it is important to maintain a holistic perspective throughout the process, ensuring a solution does not solve one problem while creating others. Furthermore, while intuition plays a role in the creative aspects of DT, over-reliance on it can result in insufficiently validated solutions. Decisions should thus ideally balance intuitive insights with solid data and research [43]. Finally, DT’s emphasis on the conceptual and ideation phases can create challenges when it comes to implementation. Organizations might lack the necessary resources, internal alignment, or logistical capacity to bring a promising DT solution to an actual product realization [44]. Yet, despite these limitations, DT still seems to be a suitable approach for the design and implementation of a user-centered mHealth application. A better understanding of the process and its distinct challenges and opportunities may, however, be needed.

### 1.4. Research Goal and Questions

Given the currently still limited adoption of DT for the development of healthcare applications, the goal of the work presented in this article is to better understand and, consequently, manifest the concrete benefits and challenges this method yields when applied to the design and implementation of an mHealth smartphone app aimed at encouraging and supporting individuals in achieving a healthy lifestyle. We report on the first iteration of this process and evaluate the insights it produced, particularly with respect to the following key design questions:

- What are the distinct needs and challenges of key users of this mHealth application (DT modes: Empathize and Define)?
- How can these needs be transferred to and implemented by an mHealth application (DT modes: Ideate and Prototype)?
- Which usability problems can be identified by a first low-fidelity prototype (DT mode: Test)?
- What can be learned form this first iteration that should be considered in follow-up design cycles (evaluation of the overall process)?

The goal of the presented study may consequently be subsumed as a structured analysis of the type of insights a DT approach produces in its first iteration when applied to the design of an mHealth application aimed at triggering behavior change in users. While the structure of the app is intended to be applicable to all components of a healthy lifestyle, in this first design iteration, we address physical activity as the initial component. We structure our report of this research endeavor as follows: Section 2 describes our methodological approach to evaluating the first iteration of using DT for the needs analysis, conceptualization, prototyping, and evaluation of a dedicated mHealth application. Section 3 reports on the results of these four phases, before Section 4 reflects on the more general insights we were able to produce from the analysis of this process. Finally, Section 5 concludes our report, addresses its limitations, and presents some future research directions.

## 2. Methodology

Our process evaluation was subdivided into four phases. The initial analysis phase (cf. Section 2.1) focused on the insights gained from the DT modes *Empathize* and *Define*, while the subsequent conception phase (cf. Section 2.2) covered learnings from the DT mode *Ideate*. Findings from the DT mode *Prototype* were produced in the development phase (cf. Section 2.3) before the evaluation phase (cf. Section 2.4) provided feedback on the DT mode *Test* and focused on the overall findings related to this first design iteration.

### 2.1. Analysis Phase

The first step of DT (i.e., *Empathize*) is usually an analytical phase, which aims at understanding users’ needs and challenges. Here, one focuses on the creation of an empathetic understanding [34], putting aside individual assumptions so as to gain true and factual insights into distinct requirements. With the given mHealth application, we followed the example of Altman et al. [33] and Roberts et al. [35] and started this analysis with a domain-specific review of the available literature, before subsequently engaging in individual brainstorming activities aimed at generating creative solution approaches. Finally, the findings from both the literature analysis and our expert creations were brought together and discussed in a workshop meeting. Next, moving to the *Define* mode, the goal was to identify and clearly specify user core needs and challenges. We used *customer journey mapping* in interdisciplinary team meetings to discuss the key elements of a user’s interaction journey and to identify problems and missing features. Then, the *comparing notes* method was used to visually sort and prioritize the collected information, and eventually produce a list of clearly defined problem statements [40].

### 2.2. Conception Phase

The DT *Ideation* mode is used to transition from a phase that focuses on problem identification to a phase that focuses on the exploration of innovative options and solutions. It is a fluctuation between focus and flare. That is, a wide area of idea generation is followed by a process of narrowing-in, in which ideas are evaluated and selected [34]. In this phase, sketching is considered an especially helpful tool, as it allows for a quick representation of a product that contains relevant features, yet does not distract with unnecessary details. The hereby often rapid cycling through various paper sketches helps to quickly recognize design lock-ins (i.e., fails) and thus produces viable solutions at a much faster pace [40,45]. Furthermore, it is an effective approach to make the ideas of different team members and stakeholders tangible and consequently triggers early feedback on potential improvements [26].

With mobile applications such as the investigated mHealth solution, sketching often includes schematic drawings of content and descriptions of user screens (e.g., wireframes). Thus, we engaged in the respective paper prototyping through ideation workshops in which we asked an interdisciplinary group of experts (i.e., physicians, medical information scientists, sports scientists, and physical therapists) to conceptualize and discuss potential application designs and their features. This type of interdisciplinary collaboration was meant to increase the evidence base and consequently improve the quality of the proposed solutions [46,47].

### 2.3. Development Phase

The output of the above-described conception phase then served as the foundation for the first, low-fidelity, platform-independent prototype of our mHealth application. We focused on an evolutionary development approach, which aims to incrementally evolve a prototype and all its parts into a final product, in contrast to the notion of a prototype representing a simple throw-away artifact [36]. In this sense, our initial prototype visualized the core idea of the envisioned mobile application. Its design was guided by principles being suggested for psychosocial intervention development and implementation [26]. Despite providing a restricted and functionally limited perspective of the system (for some parts, only portions of it), this approach was designed to collect critical user feedback, thereby facilitating the design and evaluation of potential user interactions [30].

### 2.4. Evaluation Phase

Involving stakeholders throughout the design and development process helps to distinguish between what potential technology users need and want, and what technology providers believe may be beneficial [33,48,49]. In healthcare, however, early-stage prototyping and rapid evaluation cycles are less common, as here, we face a significant tension between a potentially positive outcome and serious negative risks (e.g., healthcare failures). This often leads to reluctance, although low-stake approaches to prototyping may actually help minimize said risk and, at the same time, improve innovation [33]. In order to minimize potential risks, our mHealth prototype analyses focused on controlled, lab-based testing and feedback generation. We followed a formative study approach to explore users’ perceptions of and responses to our initial design concept [49,50]. That is, we used a combination of *Think-Aloud* (cf. Section 2.4.3) and post-evaluation interviews to explore the satisfaction, error potential, perceived first-use learnability, and cognitive load of our initial prototype [21]. Furthermore, we evaluated task completion so as to draw conclusions with regard to the effectiveness of our prototype’s functionalities.

#### 2.4.1. Participants

According to Barnum (2002) [51] and Nielsen (2012) [52], this type of formative user testing is most effective with 5–8 participants, since the cost of a greater sample size outweighs the additional insights the involvement of more users would generate. Consequently, our evaluations focused on a convenience sample of 6 potential mHealth application users. As we aimed for a rather heterogeneous sample, we screened our participants to make sure they fit the profile. The inclusion criteria for study participation were as follows: (1) the participant has no cognitive or physical impairment except for common age-related deterioration of physical (e.g., slight haptic deterioration) or cognitive (e.g., corrected vision) capabilities; (2) the participant has the ability to read German (Note: As the study was conducted in collaboration with a local health provider, the initial prototype focused on German only and not localized to other languages. The screen shots shown in Section 3 were post-edited in order to align with the language used in this article); (3) the participant adds to a certain variety of experiences using mobile applications represented in the overall sample; (4) the participant adds to a certain variety in age represented in the overall sample. Participation in our analysis was voluntary and did not involve any finical compensation.

#### 2.4.2. Procedures

Participants first received information on the overall study objective and respective research procedure and were consequently asked to once more confirm their willingness to participate by signing an informed consent document. The evaluation procedure consisted of 6 different tasks, all of which had to be completed with the given mobile application prototype on either an Apple iPhone 11 or a Samsung Galaxy S8 (Note: Since our low-fidelity prototype was able to run on both mobile operating systems, i.e., iOS and Android, we wanted to let participants select the one platform that was more convenient to them, thereby excluding potential side effects caused by a lack of platform experience). First, they had to effectively follow a theory-based process. For this initial task, there was only one possible pathway offered by the application—an initial questionnaire using ‘yes’ and `no’ buttons for navigation (cf. Figures 3 and 4). Subsequently, participants were given 5 min to obtain a basic understanding of the application, for which they were allowed to freely navigate within the app. For the second task, they had to navigate to a specific content item (cf. Figure 6a). As a third task, they had to mark a content item as favorite (cf. Figure 7a). The fourth task involved filtering content items (cf. Figure 6d). For the fifth task, they had to find more information about their individual stage of change (cf. Figure 8b), and for the final sixth task, they had to navigate to the application’s support screen. To minimize carry-over effects, we counterbalanced the order of these tasks across participants.

Participants did not receive step-by-step instructions on how to accomplish these tasks, but rather an overall task description. Nor was there a trial run, as we wanted to observe initial perceptions and responses, including feedback on first-use learnability. They were also clearly informed that they were interacting with an initial prototype aimed at clarifying requirements, which contained just the initial screens of the envisioned mobile application. Consequently, they were free to openly express all felt experiences and perceived challenges while performing the tasks and during the consecutive post-test interview. All tests were audio and screen recorded to help with subsequent data analyses.

#### 2.4.3. Think-Aloud

Participants were explicitly asked to think out loud while performing the tasks. That is, they were instructed to verbalize their thoughts and each action. For example, if they tapped the submit button, they would say out loud, *“I am tapping the submit button”*, or if they were searching for information on a screen, they would also express their thoughts throughout this search process. Participants were furthermore asked to express their thoughts while encountering problems or uncertainties. The goal of this approach was to make the participants’ thoughts transparent and, consequently, identify user-centered challenges we were unaware of.

#### 2.4.4. Task Completion

In order to evaluate participants’ effectiveness, we assessed their task completion on a binary scale. No task assistance was provided so as to not interfere with this measure. While task completion served as a relevant measure, the *Prototype* mode in DT differs from the typical pilot testing used in traditional software development projects in that it aims for exploration, rather than confirmation. With respect to our mobile application, it was thus the goal to also explore meaningful alterations to the envisioned intervention strategy [26].

#### 2.4.5. Post-Test Interview

After a participant had completed (or given up) on all tasks, he/she was asked to provide feedback in the form of a semi-structured face-to-face interview. All of these interviews took place in person at a cooperating healthcare center and followed semi-structured interview guidelines including open-ended questions such as, *“What do you mean by (…)?”* or *“Tell me more about (…)?”* or, *“Give me an example of (…)?”*, to elicit more detailed responses and clarifications. To this end, Alwashmi et al. [53] propose the matching of quantitative measures with qualitative questions. For example, a given completion rate could be matched with the question, *“What can we do to enhance the completion rate”*. Similarly, Beatty et al. [54] matched the completion time with the question, *“I noticed that the (…) feature took you longer than some of the others. Tell me more about that?”*. We decided to match our interview questions with the chosen design principles depicted in Table 1. We chose these construct categories based on the design principles suggested for psychosocial intervention development and implementation [26], i.e., satisfaction, learnability, error potential, cognitive load, and effectiveness.

All interviews were audio-recorded and, subsequently, transcribed. In addition, each session was observed, and notes were taken. Transcripts and observation notes taken during the test sessions were analyzed thematically applying framework analysis [55], which matches themes and participant responses and, thus, facilitates cross-case comparison and pattern identification.

## 3. Results

The discussions during the analysis phase (DT modes *Empathize* and *Define*) yielded the following five key features to be addressed by the envisioned mHealth application: (1) support for long-term engagement; (2) integration of individualized behavior change techniques; (3) provision of additional information on applied theories; (4) high usability so as to foster user engagement; and (5) integration of interdisciplinary experts. During the subsequent conception phase (DT mode *Ideation*), these needs-based requirements were matched with concrete analytical, as well as practical solution approaches. That is, the needs for long-term engagement and individualized behavior change were addressed by transparently integrating a transtheoretical model of change via a staging algorithm into the mHealth application workflow (cf. Section 3.1). As for the potential usability issues and the integration of interdisciplinary experts, it was found that the DT method would need to more strongly engage in an iterative, user-centered creation process, where feedback from multiple stakeholders is integrated at all stages of development (cf. Section 3.2 and Section 3.3).

### 3.1. Conception: Embedding a Theory of Change into mHealth

In order to tackle long-term engagement and support behavior change, we suggested to embed a health theory into the feature set of our envisioned mHealth application. Change theory, in general, holds that behavior change is a complex process, and that multiple actions and adaptations over time are required to sustain health behavior change. Thus, intervention programs that are designed for individuals who are ready to change their behavior may be rendered ineffective or even detrimental for those who lack this type of intrinsic motivation [17]. Individualized content and personalization, on the other hand, can significantly increase the effectiveness of such interventions [18]—the goal being to not only help a small number of individuals improve their health once, but rather, to help a larger number of people achieve and maintain such positive health behaviors over a longer period of time. To this end, the most widely used models for behavior change interventions are the Health Belief Model (HBM) and the Transtheoretical Model of Change (TTM) [56,57]. The HBM [58] is primarily concerned with the reasons why people do or do not use preventive services and analyzes their beliefs and perceptions of the benefits and barriers of changing their behavior. The TTM [59] also includes these constructs, but offers valuable advantages for long-term changes in health behavior, for which we found it would better suit our analysis.

#### 3.1.1. The Health Belief Model

The HBM [58] is a psychological framework that explores why people adopt healthy behaviors. It suggests that people are more likely to change their habits if they feel personally susceptible to a serious health threat, believe the benefits of change outweigh any downsides, see few barriers to action, are exposed to cues prompting action, and feel confident in their ability to succeed. The HBM helps healthcare professionals tailor interventions to increase healthy behaviors, promoting positive health outcomes. Originally developed in the 1950s [60], the HBM has substantial empirical support and remains a widely used tool to promote positive health outcomes [17]. Due to its strong reliance on people’s individual belief system, which is often influenced by external factors and psychological characteristics, HBM’s practicality to underpin an mHealth application seems, however, rather limited.

#### 3.1.2. The Transtheoretical Model of Change

**Figure 2:**
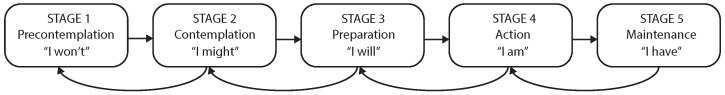
The 5 stages of the Transtheoretical Model of Change.

The TTM [59] is another commonly applied models in health-related behavior change [17,61]. It describes a cyclical pattern of movement through different stages of change, i.e., precontemplation, contemplation, preparation, action, and maintenance (cf. Figure 2). The temporal dimension of progressing through these stages is important, underlining that changing behavior is not a one-off event, but rather a process that takes time. In each of these stages, appropriate processes and principles of change are systematically integrated so as to optimally support the progression from one stage to the next [59,62].

During precontemplation, people usually have no intention to change due to a lack of information concerning potential consequences of their current behavior [63] or because they have already undergone unsuccessful change attempts [64]. People in this stage tend to avoid thinking, reading, or talking about their currently risky or unhealthy behavior. Negative aspects of their current (unchanged) behavior are not experienced very emotionally. They devote little energy and time to reevaluating themselves and, thus, appear rather resistant towards health promotion programs [65]. During the proceeding contemplation, people become attentive to change their behavior [66]. They become aware of their unhealthy behavior and the benefits change may provide, and thus start to seriously consider problem resolution. However, they lack clear guidance so that taking action often seems cumbersome. This is where people usually feel stuck, overwhelmed by a lack of understanding the costs and benefits of change. The first small steps and their evaluation during the subsequent preparation stage helps them control the situation and, consequently, supports them in withdrawing from their old behavior [67]. The action stage then stands for modified behavior with a considerable commitment of time and energy. People in this stage have taken action regularly, although there is no certainty that they will retain this behavior [68]. Higher levels of self-liberation and believing in one’s autonomy to change one’s life are typical for this stage. It is the most visible stage and, thus, receives the greatest external recognition. Reaching it, however, does not imply that behavior has been changed sustainably. It is only the maintenance stage where the new behavior has become routine and, consequently, embedded into an individual’s lifestyle. Although, from time to time, it requires preventive actions so that a potential relapse to an earlier stage is circumvented [69].

#### 3.1.3. A TTM-Based Staging Algorithm for mHealth

Each of the above-outlined stages benefits from supportive measures, ranging from guidelines to dedicated intervention programs. And this is where an mHealth application may set its focus. For example, during the precontemplation stage and contemplation stage, features that trigger awareness raising, dramatic relief, and environmental reevaluation can help in progressing to the next stage. This can be achieved by proactively providing relevant information on potential problems and the respective benefits an intervention may offer. For the stages of preparation, action, and maintenance, however, the focus should be on behavioral techniques, rather than experiential processes of change. More action-oriented programs are likely to be successful, for example by countering sedentary habits with healthier ones. In addition, supportive strategies to improve self-efficacy become more relevant.

**Figure 3:**
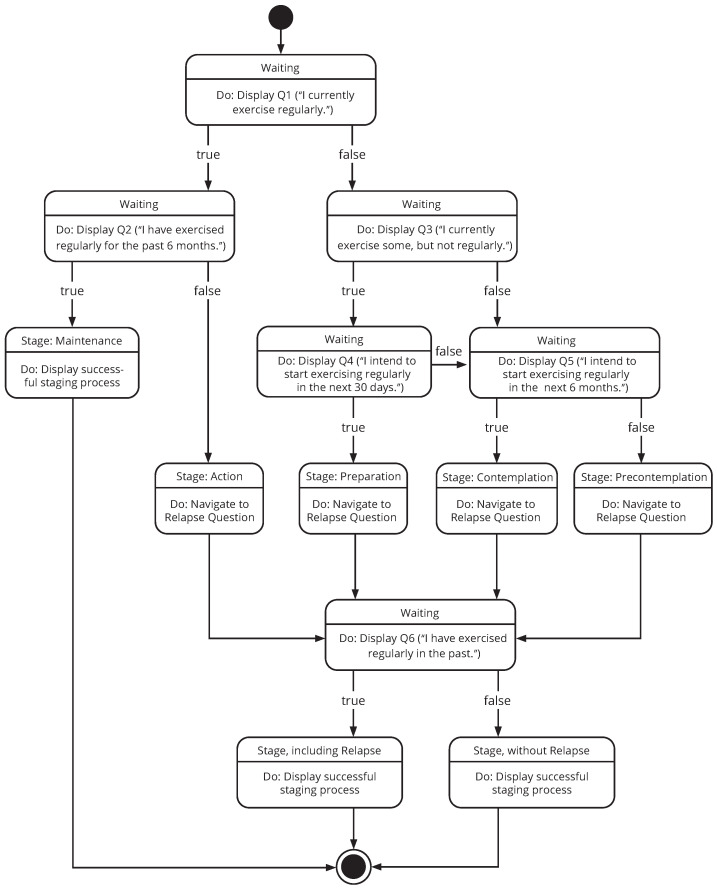
Visualization of a TTM-based staging algorithm.

Before integrating the measures of the individual stages of change, the initial step requires an assessment of the current stage of behavioral change. The results of our conception phase thus converged into the proposal for a well-defined staging procedure to be integrated into the mHealth application. The proposed procedure is based on TTM guidelines and lessons (cf. [59,61,62,70,71]) and adapted to be used in the given mHealth setting in that it uses concrete questions to guide a user through the process (cf. Figure 3).

The initial question (Q1) aims to define a person’s current status (*“I currently exercise regularly.”*). If the answer to this question is *“Yes”*, a subsequent question (i.e., Q2: *“I have exercised regularly for the past 6 months”*) aims to determine whether a maintenance intervention (answer: *“Yes”*) or an action intervention (answer: *“No”*) should be triggered. On the other hand, if the answer to Q1 is *“No”*, a follow-up question (Q3: *“I currently exercise some, but not regularly”*) aims to define whether a person has a basic openness towards a potential behavior change. Depending on this answer, the next question tries to determine the level of commitment towards change that can be expected. That is, if Q3 is answered with *“Yes”*, a subsequent question inquires about a person’s plan for the next 30 days (i.e., Q4: *“I intend to start exercising regularly in the next 30 days”*); if the answer to Q3 is *“No”*, the inquiry focuses rather on the next 6 months (i.e., Q5: *“I intend to start exercising regularly in the next 6 months”*). Based on these answers, we then see either a preparation (answer Q4: *“Yes”*), a contemplation (answer Q4: *“No”* and subsequent answer to Q5: *“Yes”*), or a precontemplation (answer Q4: *“No”* and subsequent answer to Q5: *“No”*) action to be triggered. Subsequently, the final question (i.e., Q6: *“I have exercised regularly in the past”*) periodically evaluates whether somebody experiences a relapse (answer: *“Yes”*) or keeps up with the changed behavior (answer: *“No”*).

### 3.2. Development: Implementing an Initial Prototype

Based on the insights and respective feature proposals developed in the conception phase (cf. Section 3.1), we implemented an initial prototype of the mHealth app using using React Native v0.69 (Online: https://reactnative.dev/ [accessed: 16 February 2024]) and the Expo Go SDK v45, (Online: https://expo.dev/ [accessed: 16 February 2024]). It consists of five screen units, i.e., the initial *Staging Process*, *Content*, *Favorites*, *Stages*, and *Info*. Each screen unit, except for the *Staging Process*, is subdivided into several sub-screens and contains a header, a main part, and a footer navigation. Headers include a sub-screen title displaying the active sub-screen, and a clickable icon or text on the right side to allow for navigating to another sub-screen. The main parts show specific content according to the active sub-screen, and footers allow for navigating between entire screen units. The *Staging Process* screen unit contains different sub-screens as well, yet it does not offer header or footer navigation as it represents the initial start of the app. All user interactions (i.e., tapping on buttons, header icons, menu icons, toggle switches, or content cards) offer optical feedback. Despite some system-dependent differences in the navigation and header sections, screen units and sub-screens look similar in both the iOS and Android application.

#### 3.2.1. The Staging Process Screen Unit

**Figure 4:**
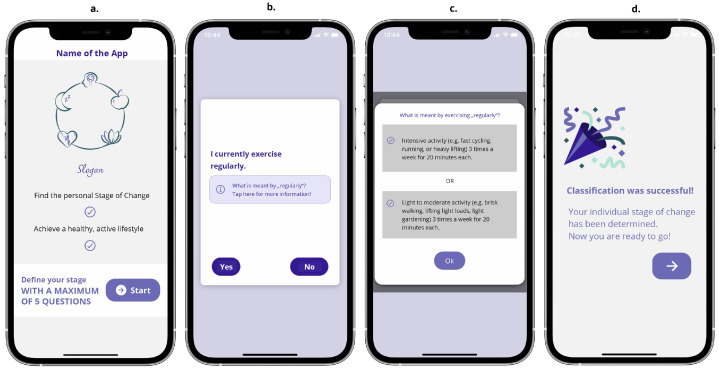
Sub-screens of the *Staging Process* screen unit: (**a**) landing screen, (**b**) example question of staging algorithm, (**c**) example explanation of the term “regularly”, (**d**) screen displaying the success of the staging process.

The initial screen of the *Staging Process* acts as the landing page of the app, showing compact information about its content and then leading directly to the earlier-outlined staging process. The herein-implemented staging algorithm (cf. Section 3.1.3) guides the user through a maximum of five questions. Relevant explanations are offered via dedicated information buttons. Following this process, the user eventually reaches his/her individual *Stages* screen unit (cf. Figure 4).

#### 3.2.2. The Stages Screen Unit

**Figure 5:**
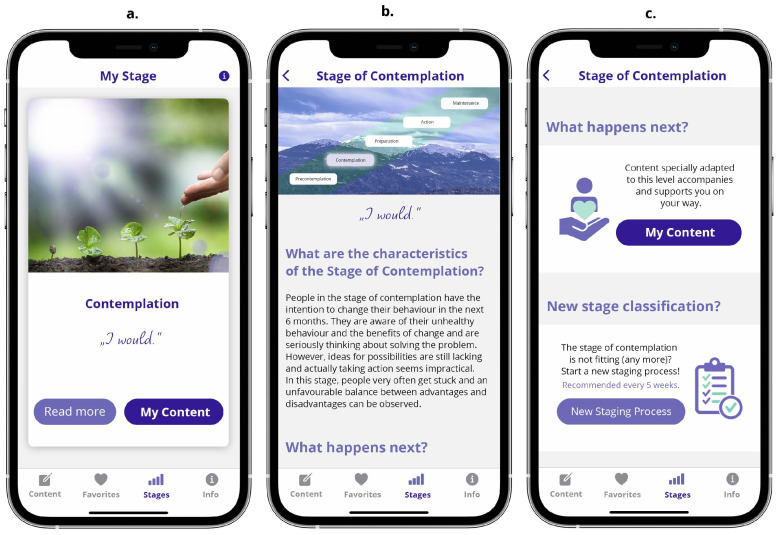
Sub-screens of the *Stage* screen unit: (**a**) stage overview, (**b**) stage details, (**c**) stage details continuation (vertically scrolled).

The *Stages* screen unit starts with an overview of the result from the staging process. The user then has the opportunity to either tap on a button to read more about his or her stage or tap on another button navigating immediately to his or her content (i.e., the *Content* screen unit). The former offers written content explaining an individual’s stage of change, whereas from the latter, the user can navigate to his or her content or scroll down to additional information about conducting a new staging process (cf. Figure 5).

#### 3.2.3. The Content Screen Unit

**Figure 6:**
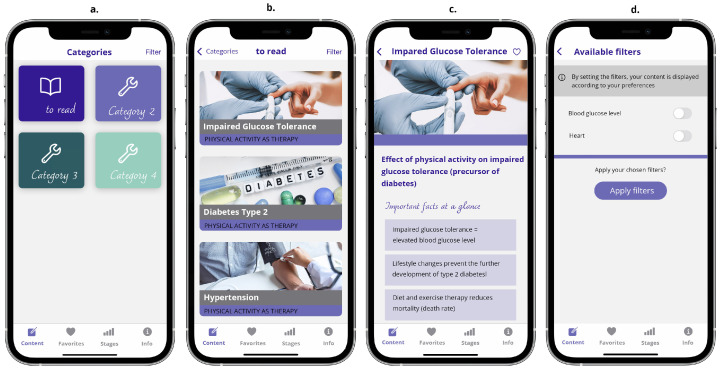
Sub-screens of the *Content* screen unit: (**a**) content categories, (**b**) content of category *to read*, (initial part of a scrollable view), (**c**) details of example content *Impaired Glucose Tolerance*, (initial part of a scrollable view), (**d**) filters screen.

The *Content* screen unit is where categorized and individualized content (e.g., specific techniques for each stage of change) is displayed. Currently, it only holds dummy data for one category to test the application’s basic functionality. It is planned, however, that the *Content* unit will become the landing page of the application after a user has gone through the staging process. Starting at an overview screen displaying available categories, a user can then navigate, select, and filter different types of content (cf. Figure 6).

#### 3.2.4. The Favorites Screen Unit

**Figure 7:**
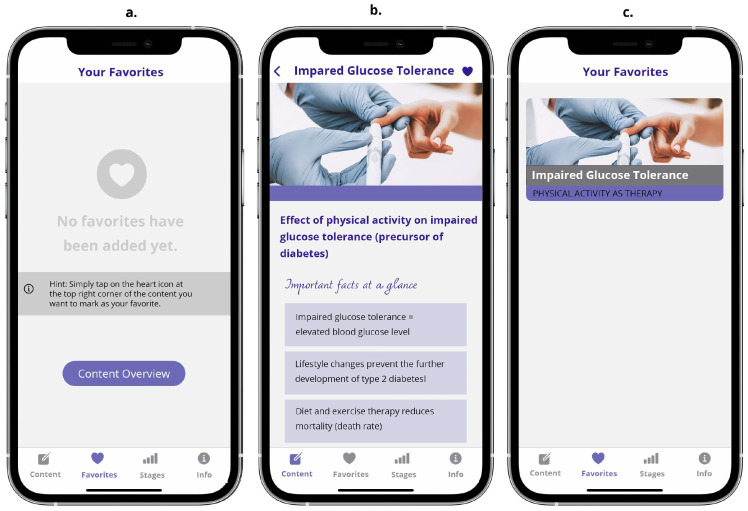
Sub-screens of the *Favorites* screen unit: (**a**) initial screen when no content is marked as a favorite; (**b**) marking content as a favorite (filled heart icon on right side of the header after tapping on it); (**c**) marked content is now stored as a favorite.

The *Favorites* screen unit allows the user to mark content as his or her favorite. Tapping on the heart symbol, the selected content will automatically be added to the *Favorites* screen. In case no favorite content has so far been marked, the screen displays information on how to add favorites (cf. Figure 7).

#### 3.2.5. The Info Screen Unit

**Figure 8:**
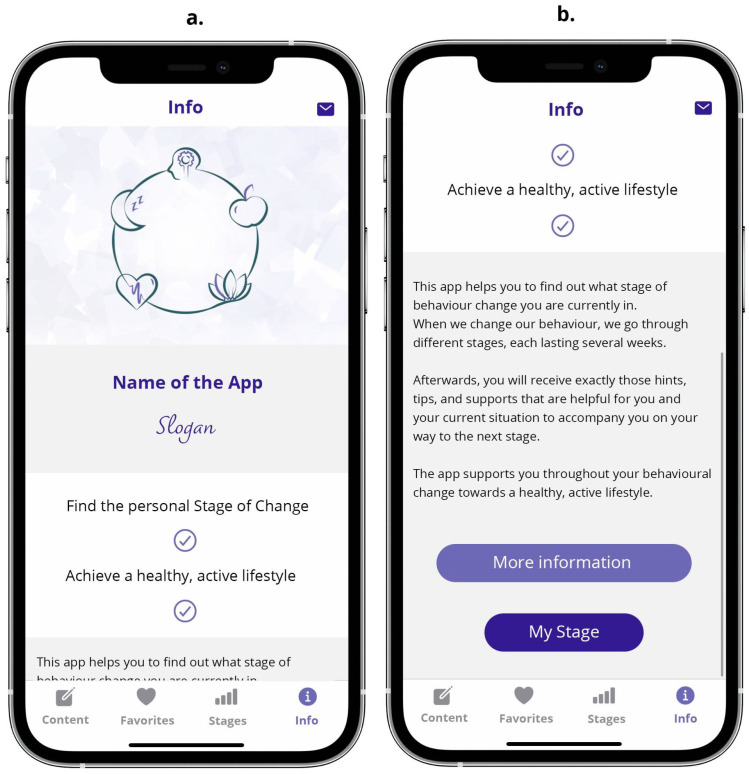
Screens of the *Info* screen unit: (**a**) first screen of Info unit (initial part of a scrollable view) displaying basic information, (**b**) additional part of the scrollable view including buttons to navigate to a screen displaying more detailed information or to the *Stage* screen unit.

Finally, the sub-screens of the *Info* screen unit offer additional information about the application, as well as a contact form. The *Info* screen unit can be reached from the bottom of any other screen by tapping on the information circle icon in the footer navigation (cf. Figure 8).

### 3.3. Evaluation: Collecting Feedback from Six Potential mHealth Users

As outlined in Section 2.4.1, we exposed six potential mHealth users (P1–P6) to the above-described prototype. One challenge of DT in healthcare lies in the difficulty of capturing the stories of outliers through conventional health research methodologies, which typically emphasize statistical analysis of large sample sizes to generate results that can be generalized [33]. Mixed-methods approaches, on the other hand, combine qualitative and quantitative research elements to increase the breadth and depth of researchers’ understanding and corroboration [72]. To this end, Altman et al. [33] recommend using a qualitative DT approach, including usability tests with small groups of the target users, in the early stages of a development process. In order to generate insights regarding the key needs of a broad target population, it is furthermore suggested to recruit a diverse group of participants. In our case, this meant introducing variability so as to ensure that participants vary in terms of age groups and experience with mHealth applications. Table 2 provides some characteristics of the study participants we were able to recruit for this investigation.

Our participants were asked to complete six different tasks (cf. Section 2.4.2). The distinct goal of this initial evaluation was to collect feedback on people’s perceptions as to (1) how well the prototype concept would satisfy their needs concerning behavior change; (2) how learnable they believe the presented concepts are; (3) what errors they would run into; (4) how they would cope with the cognitive load triggered by the interaction; and (5) how effective those interactions would be.

#### 3.3.1. Satisfaction

Overall, participants seemed to be very satisfied with the presented prototype concept. They found the offered features valuable and pleasant to use. The staging process at the beginning of the interaction received particularly positive feedback, as can be seen from the following participant comments:

*The staging process was great. It was easy - not time-consuming. I was surprised how fast I accessed the app content. That was pleasant, because I don’t like to answer too many questions.*

[P4]

*[After the staging process] Wow, I am already done with the questions. Now I am ready to go. (…) It is easy to understand.*

[P1]

*The image is very nice [Icon after staging process]. I think that’s good. People like that too, the feeling that you’ve achieved something.*

[P5]

*I like that I have an immediate overview and that I can still filter and see exactly what I am interested in.*

[P2]

*I believe that it helps people when they are given something to take with them [application] that helps them along the way.*

[P6]

Although some participants expressed ideas for additional features such as a dedicated user profile or dark mode, participants highlighted that these features may be added at a later stage. One participant [P4] mentioned that it would be easier for her to navigate through the app if she had one central point of information, i.e., a user profile, as she is used to from other apps.

*There is one comfort thing - Dark Mode. (…) But I wouldn’t force it, but leave it open to chose. Some people prefer the light version.*

[P3]

*I’m just missing a user profile yet. I always want to see everything at a glance.*

[P4]

#### 3.3.2. Learnability

None of our participants felt the need for technical support in order to use the system. On the contrary, they found it easy to build an understanding of the prototype’s conceptional model. In particular, the clear design and self-explanatory structure were appreciated. Rapidly building an understanding of the app’s use was provided by offering different forms of information throughout the app, beginning with short versions only consisting of a few words or icons, and becoming more detailed for users struggling to understand it, or just being more interested. This structure was well received from participants and is consistent with the findings that exhaustive documentation is generally experienced as burdensome, resulting in the fact that most users do not read manuals.

*I think the menu at the bottom is comfortable. I find it convenient. You can see it right away.*

[P4]

*It’s actually really easy to use. It is very self-explanatory.*

[P2]

*Really simple, even for me - because I’m actually not an app person at all.*

[P1]

Only one participant stated that he would need more time to completely understand the application. The post-test interview (cf. Section 2.4.5) revealed, however, that this lack of understanding was based on the false belief that the presented prototype would be an actual final product. Although a more feature-complete app would probably mitigate such concerns, both the literature (cf. [54]), as well as statements from the post-test interview with the oldest participant suggest that an additional tutorial or in-person training would significantly boost first-use learnability.

*I would have to take a little more time - I have already noticed that. (…) Yes, a tutorial certainly adds to the simplicity.*

[P6]

#### 3.3.3. Error Potential

While there were no difficulties or errors reported during the post-test interviews, an analysis of the Think-Aloud protocol (cf. Section 2.4.3) revealed a number of minor issues. For example, one participant tapped on *“Filters”* when actually trying to mark content as favorite. Another participant tapped on *“Stages”* when actually trying to navigate to specific content. Yet, both of them immediately realized their mistake and were consequently able to quickly find the correct action.

*Even if you tap somewhere else, it does not matter - you just go back.*

[P2]

*If someone does not want to do a new staging process, but has already tapped the button, there is a hint [Alert] - I think that’s good.*

[P5]

The two most experienced participants, [P2] and [P5], assumed that the back button on the filters screen would immediately save changes after they had been applied. Yet, with the current prototype, changes are only saved if a user taps the button *“Apply Filters”*. Although the two participants immediately realized and corrected their mistake, revealing these types of problems by including lead users earlier in the design process would have helped improve the effectiveness of the initial prototype [73].

#### 3.3.4. Cognitive Load

According to a meta-analysis by Van Genugten et al., Internet-based health behavior change interventions are more effective when they take little time to understand and, thus, require little cognitive load [74]. From the observations and feedback we received, it seems that our prototype interactions induced rather low cognitive load. Participants appreciated the simplicity of the prototype’s structures and functionalities, and they positively mentioned the overview, which was provided on each screen, and that further information could be gathered by navigating to a separate information screen, thereby reducing the potential for clutter on the main screen.

*I find it very advantageous that there is not too much input at once. Everything is clearly arranged on one page and it’s easy to find everything.*

[P1]

*I like that there are only four items here [main menu / bottom tab navigator]. Then it is not so cluttered. Also the icons are well done - you recognize right away what it is about.*

[P5]

*You just have to try it out a bit. (…) So I think it’s applicable - even for people who don’t have that much experience with mobile phones.*

[P6]

Also, the use of familiar icons (e.g., the heart icon to mark content as favorite) helped users to easily find and recognize what they were looking for.

*With the favorites, for example, it’s also good that it’s a heart - then it’s like people have it in their minds.*

[P5]

#### 3.3.5. Effectiveness

In particular, in the early stages of development, a mixed-methods approach suggests focusing most of the assessments on qualitative data [53]. Consequently, we chose post-test interviews as a data-collection method (cf. questions on the design principle `Effectiveness’ in Table 1). However, following the recommendations of Alwashmi et al. [53] and Beatty et al. [54], we also incorporated an additional quantitative assessment. We wanted to ensure the evaluation of whether users are generally capable of completing the tasks, and so, we used a binary scale to determine whether a task was successfully completed or not. The effectiveness of the task dealing with the staging process (i.e., the first task) was particularly important to us. As we newly developed this digitalized staging process and its associated interfaces, we wanted to determine whether users could successfully complete this process. Our results showed that all six participants were able to complete all six tasks (100% completion rate), and additional qualitative findings furthermore indicated that users were particularly surprised by how fast this process was completed. The remaining five tasks addressed the structural constructs within the application into which content based on the current stage of behavioral change was integrated. Also, here, our results showed that all six users were able to navigate to specific content (i.e., second task), mark it as a favorite (i.e., third task), filter content (i.e., fourth task), find more information about their individual stage of behavioral change (i.e., fifth task), and discover support options within the app (i.e., sixth task).

## 4. Discussion

Next, we will reflect on the more general insights this first DT iteration, driven by the phases analysis (DT stages Empathizing and Defining; cf. Section 2.1), conception (DT stage Ideating; cf. Section 2.2), development (DT stage Prototype; cf. Section 2.3), and evaluation (DT stage Test; cf. Section 2.4) has produced.

### 4.1. The Importance of Theory

Our analysis phase has particularly highlighted that, for mHealth interventions to be effective, it is crucial for them to address the need for long-term engagement. Although there has been earlier evidence that interventions with a theoretical basis support long-term engagement (e.g., [18,23,25]) and that this makes them more effective [17,18], most mHealth applications lack this integration of health behavior theory [15,26,75]. This is surprising since the required personalization and content optimizations are greatly supported by these types of applications, allowing for an easy mapping between interactions and individual data and responses. According to the TTM, behavior change techniques cannot be successfully applied without this linking, as mismatching a user’s current stage of change may render a respective intervention ineffective or even detrimental [62]. Especially for interventions applying certain behavior change techniques, the implementation of theory-driven features and content, therefore, seems not only crucial, but, rather, imperative [76]. Furthermore, since changing behavior is known to follow a cyclic pattern, including relapses to lower stages of change, interventions focusing solely on the end goal, or on a linear progress through stages, are likely to lead to disappointing and discouraging results. Efficient change depends on performing the right action at the right time. So-called *self-changers*, however, rarely take the time to assess their current stage and then tailor the processes in a conscious and meaningful manner [62]. This further highlights the potential mHealth applications can offer in guiding users through a conscious behavior-change process.

Unfortunately, however, even those mHealth application that embed a theoretical underpinning, such as goal-setting or self-monitoring, rarely evaluate their effectiveness [15]. That is, their use is quickly explained, yet no details on their actual performance are provided [75,77]. To this end, an analysis by Middelweerd et al. [78] showed that integrated behavior-change techniques are often ineffective and thus require improvements. Also, other reviews investigating behavior change theories in mHealth applications point to a lack or inadequate use of theoretical constructs (e.g., [75,77,79]). So, it seems that, despite the rapid development of mHealth technologies, which hold great potential to prevent diseases, improve treatment, increase access to health services, and reduce healthcare costs, mHealth research and the integration of the respective change theories are lacking [80].

Those mHealth applications that currently incorporate behavior-change techniques are mainly designed to address content for users who are in the stage of preparation (e.g., those who are already physically active, but not on a regular basis) [81]. It is furthermore notable that technologies aimed at encouraging behavioral change often adhere to a “one-size-fits-all” approach, meaning that all users receive the same techniques and the same feedback, regardless of their stage of behavior change [82]. Sadly, this approach overlooks certain groups of individuals, i.e., those having no intention to change their behavior or having lost their intention (stage of precontemplation); or those who are willing to change, but are not able to take action and bridge the intention–behavior-gap [83] (stage of contemplation). Ferron and Massa [81], as well as Lee et al. [84] thus highlight the importance of mobile applications offering varying content based on the TTM. Furthermore, it needs to be noted that the cyclical pattern of a change process [85] implies that individuals do not only remain at a constant stage or ascend to a higher stage, but often also experience relapses to lower stages. An application that focuses solely on static stage-specific content is thus likely to have limited success. Consequently, there is a need for the ability to deliver variable content.

Although there are studies that mention that they are using stage-of-change-based content for their application users (e.g., [86,87,88]), there are, to the best of our knowledge, no studies that have integrated the staging process inside of the application, or focused on the design of a mobile application applying the TTM as a basis for dynamic stage-specific content. The unique feature of our application prototype thus lies in this implementation of a TTM-based algorithm, along with its corresponding interfaces, into a structure that may serve as a basis for the future integration of dynamic change-process-stage-based content.

### 4.2. The Importance of Processes

Next to theory embedding is the design and development process, which often impacts the adoption and consequent success of mHealth applications. According to Glanz and Bishop’s review on the role behavioral science theory plays in the development and implementation of public health interventions [17], it may be argued that the success of such solutions is greatly improved by participatory, user-centered processes. Our initial analysis has confirmed this need for user engagement, which is usually coupled with a solution’s usability needs [28,29]. To this end, Beatty et al. [54], for example, found that iteratively revising an mHealth application for cardiac rehabilitation resulted in significant usability improvements. Historically, however, technology-supported evidence-based treatment has primarily focused on robust and often complicated solutions for highly specific problems. The emphasis was on engineering (i.e., building functional solutions meeting technical specifications) rather than designing (i.e., ensuring ease of use, aesthetics, and parsimony meeting requirements in compelling ways) [26].

While traditionally, designers have focused on the look and feel of products, process-driven frameworks, such as DT, have recently become a solution approach to many complex problem spaces, including healthcare [42]. It is the cyclic nature of these frameworks in, running iteratively through analytical, practical, and evaluative phases, in particular that allows for the flexible and holistic addressing of complex, often changing user problems [26]. The analytical phase of DT is thereby characterized by a thorough problem understanding, focusing on underlying causes [41], while leaving little room for assumptions [40]. In other words, before engineers can solve problems, designers should help discover them. An ideation stage, in which various product stakeholders are invited to iteratively discuss and redesign initial prototypes, not only allows for the integration of ideas, but also supports the identification of potentially costly problems. On the other hand, to evaluate components, rapid prototypes in the form of so-called micro-trials may provide important feedback for reviewing and discussing design decisions [89]. Here, the aim is not to focus on full prototype evaluations, but, rather, on specific small-scale factors and outcomes. And, in an early design stage, like with our mHealth application, the micro-trials concept can also be used to evaluate the extent to which potential end-users find an intervention component appropriate and acceptable.

### 4.3. The Importance of Users

Working with end-users at the early stages of the design process is crucial in user-centered design, especially in healthcare [49,50]. mHealth applications have to be designed for diverse user groups having various characteristics, capabilities, and needs to achieve the identified goals [30,90]. To increase accessibility, we focused on working with a heterogeneous user group; i.e., a (small) sample representing a population with different age groups and various experiences using mobile (health) apps. The impact of end-user diversity is often underestimated by product developers, who tend to base their design concepts on people similar to themselves [91]. Yet, only through integrating diverse user groups is it possible to move beyond generic user models and, consequently, towards a more careful identification and nuanced understanding of the users’ actual desires and needs [26].

The inclusion of particularly advanced users (lead or extreme users) has the potential to improve the efficiency of the design process [73]. The d.school Design Thinking toolkit [34] thus suggests to work with extreme users so as to reveal meaningful needs and observe work-arounds. Even if some of these extreme users’ needs may be too advanced and not as relevant for less experienced users, combining them with other feedback allows for a more holistic understanding of the problem space [90].

Finally, asking users to think out loud as they perform given tasks helps to better understand their thoughts and needs. Additionally, following up on interesting comments, movements, and pauses can be helpful as well [34].

### 4.4. The Importance of Interdisciplinary Teams

Focus group data show that people using health applications express concern about whether the technology stems from a legitimate and reputable source and if the herein provided information and advice is accurate and safe. Applications that have included domain experts in their development are considered preferable and more persuasive [9]. So, there is a great need for collaboration with interdisciplinary experts, particularly when designing mHealth applications. Still, reviews show that most solutions targeting health behavior change are not developed in collaboration with academics or health professionals and that the content is usually not aligned with clinical guidelines and evidence-based practices [75,77,78,92]. Cowan et al. [75] thus suggest that there is an enormous potential in fostering the collaboration between health behavior experts and application developers in order to improve software towards better health outcomes. Middelweerd et al. [78] share this view and suggest collaborations between developers, health professionals, and behavior-change experts in order to enhance health promotion.

The combination of several intervention components like counseling sessions or physical education (multi-component interventions) leads to significant improvements in behavioral and health outcomes [8]. Especially for long-term health behavior change, multi-component interventions have been shown to be superior to stand-alone interventions [25,93]. Yet, the inclusion of healthcare providers is vital to optimize the integration of intervention components. Beatty et al. [54], for example, found that patients expect to share their data with providers and that this sharing aspect has the potential to positively influence user satisfaction. Other studies also point to this positive impact patient–provider communication may have on user satisfaction [54,94]. Also, one of our study participants highlighted that it would be advantageous to have the possibility to directly contact a (healthcare) provider.

Many healthcare issues exist across different disciplines and sectors, dealing with heterogeneous user groups [35,95]. This is why Roberts et al. [35] suggest radical collaboration as one of the vital aspects of DT for healthcare. Although it may be challenging to find time-slots that would allow for the integration of healthcare professionals into co-creation and feedback sessions, it is likely to pay off in the long run, for example if the final product then significantly improves the efficiency of the interaction between a healthcare professional and a patient [18,37].

To this end, the cooperation with a healthcare center would furthermore offer the opportunity to not only reach individuals already in the stage of contemplation (already having some intention to change their behavior), but also those in the stage of precontemplation who currently are not at a point where they intend to change their behavior.

## 5. Conclusions, Limitations, and Future Outlook

The work presented in this article focused on the development process for a theory-based, human-centered mHealth application aimed to encourage and support individuals in achieving a healthy lifestyle. As part of this work, we also created, integrated, and evaluated a staging algorithm based on the Transtheoretical Model of Behavior Change. It forms a useful basis for individualized techniques to consider the actual needs of users trying to change their behavior. Investigating the process hereby showed that it is important to follow a structured, design-driven approach so as to achieve high usability and early end-user engagement, and that collaborating with interdisciplinary experts may significantly help in identifying potential problems and challenges that may hinder the effectiveness of an envisioned mHealth app.

Yet, several limitations to these findings need to be considered. On the one hand, our sample size for the evaluation of the mHealth app was very small and, consequently, prevents us from drawing more general conclusions. Although we managed to have representatives from six different age groups and various experiences in the use of (mHealth) applications, future work would need to include more perspectives and thereby particularly emphasize the diversity of possible factors associated with the use of mobile technology for health behavior change. On the other hand, additional verification of the proposed staging algorithm is required. Thus, additional design iterations with end-users should aim at determining the effectiveness of the different processes of change, particularly with respect to long-term engagement. Finally, future work should also look at relevant changes concerning health policies and regulations, such as the upcoming European Health Technology Assessment Regulation (Online: https://health.ec.europa.eu/health-technology-assessment/regulation-health-technology-assessment_en [accessed: 24 July 2023]), which may have a significant impact on the uptake, support, and continued use of mHealth applications.

## Figures and Tables

**Table 1:** Post-test interview questions and construct matching.

Design Principle	Questions
Satisfaction	*“How did you find the integration of various functions in this app?”* *“How can we make it better?”* *“Would you need the support of a technical person to be able to use this system?”* *“How would you contact them: phone, email, or messaging?”*
Learnability	*“How did you learn to use the app?”* *“How can we reduce the time it takes to learn the app?”*
Error Potential	*“Did you have any troubles when using the app?”* *“Where?”* *“How can we fix it?”* *“What can we do to help users avoid the same error?”*
Cognitive Load	*“How do you feel about the complexity of the app?”* *“How can we simplify it?”* *“Do you have any recommendations to make the wording and interface easier to use?”* *“How did you feel about the consistency of the app?”* *“How can we simplify it?”’*
Effectiveness	Depending on the observed effectiveness of a user: *“I noticed that you did not complete the task (…)”* *“Other users were struggling with completing the task (…)”* *“What can we do to enhance the completion rate?”*

**Table 2:** Characteristics of study participants.

No	Age Group	Weekly App Use	Types of App Use	Health App Use	Future Health App Use
P1	35–44	4–9 h	Communication Social Media News/Information Education	No	Yes
P2	55–64	4–9 h	Communication Social Media	No	Yes
P3	18–24	>40 h	Communication Social Media Games/Entertainment News/Information Education Lifestyle	Yes (Fitness apps)	Yes
P4	25–34	10–19 h	Communication Social Media News/Information Education Lifestyle Utility/Productivity	Yes (Gymodo)	Yes
P5	45–55	20–40 h	Communication Social Media Games/Entertainment News/Information Education Lifestyle Utility/Productivity	Yes (Yazio, Fitnesspoint)	Yes
P6	>65	10–19 h	Communication Social Media News/Information Education Utility/Productivity	No	Yes

## Data Availability

Data are contained within the article.

## Abbreviations

The following abbreviations are used in this manuscript:

DT	Design Thinking
HBM	Health Belief Model
NCD	Non-communicable diseases
TTM	Transtheoretical Model of Change
UCD	user-centered design
WHO	World Health Organization
